# The ATP bioluminescence assay: a new application and optimization for viability testing in the parasitic nematode *Haemonchus contortus*

**DOI:** 10.1186/s13567-021-00980-4

**Published:** 2021-09-30

**Authors:** Linh Thuy Nguyen, Markéta Zajíčková, Eva Mašátová, Petra Matoušková, Lenka Skálová

**Affiliations:** Department of Biochemical Sciences, Faculty of Pharmacy, Charles University, Heyrovského 1203, 500 05 Hradec Králové, Czech Republic

**Keywords:** optimized protocol, levamisole, anthelmintics, helminths, adult worms, exsheathed third-stage larvae

## Abstract

The parasitic gastrointestinal nematode *Haemonchus contortus* causes serious economic losses to agriculture due to infection and disease in small ruminant livestock. The development of new therapies requires appropriate viability testing, with methods nowadays relying on larval motility or development using procedures that involve microscopy. None of the existing biochemical methods, however, are performed in adults, the target stage of the anthelmintic compounds. Here we present a new test for the viability of *H. contortus* adults and exsheathed third-stage larvae which is based on a bioluminescent assay of ATP content normalized to total protein concentration measured using bicinchoninic acid. All the procedure steps were optimized to achieve maximal sensitivity and robustness. This novel method can be used as a complementary assay for the phenotypic screening of new compounds with potential antinematode activity in exsheathed third-stage larvae and in adult males. Additionally, it might be used for the detection of drug-resistant isolates.

**Supplementary Information:**

The online version contains supplementary material available at 10.1186/s13567-021-00980-4.

## Introduction

Infections with gastrointestinal parasites cause huge problems in ruminant production, health, and welfare worldwide. *Haemonchus contortus* (barber’s pole worm) is one of the major parasitic nematodes of small ruminants. This worm accounts annually for great economic losses and poses a threat to the productivity of farmers [1]. The *H. contortus* life cycle is direct; adults feed on blood, causing anaemia and chronic inflammation. Despite efforts for the sustainable control of this parasite and related ones, treatment relies heavily on the use of chemical anthelmintics. However, their effectiveness is limited due to increasing drug resistance in nematodes, including *H. contortus*, therefore an extensive search has been underway for novel anthelmintics [2]. Although several studies using the primary screening of various compound libraries as well as drug repurposing have revealed hit compounds [3], no new anthelmintics have been introduced to the market since monepantel (MOP), the latest amino-acetonitrile derivative discovered in 2008 [4]. Even so, the development of new therapies requires appropriate methods. Nowadays, drug effect is in the majority assessed by observing the motility or development of larvae through processes which involve microscopy or other monitoring systems [5, 6]. While these methods allow the high-throughput assessment of anthelmintic drug efficacy, they have many limitations. The most significant drawback of all commonly used methods testing the activity of potential anthelmintics is that the tests are not performed at the parasitic stages living inside a host (L4 larvae and adults) against which anthelmintics should act. At present, only motility and/or feeding studies based on radiolabelling on adults are available [7, 8].

Taking a broader view, drug discovery and development often include testing cytotoxicity. These assays are used for measuring some aspects of general metabolism or an enzymatic activity, with methods often utilising colorimetric or fluorometric detection [9]. In choosing a method that might be adapted to nematodes, the concept of colorimetric assays would be dependent on the absorption of the reagent by a viable worm which, to our knowledge, has not been proven. Moreover, exposing cells to tetrazolium reagents or resazurin redox indicators has been demonstrated to be toxic to cells [10]. Hence, we decided to circumvent these limitations by choosing a viability marker—adenosine triphosphate (ATP).

ATP is formed exclusively in the mitochondria and is universally seen as the energy exchange factor which fuels the processes of motile contraction, phosphorylation, and active transport [11]. The best method to measure intracellular ATP is using the firefly luciferase. A well-established technique for testing viability in a variety of cell cultures, the ATP assay utilizes ATP dependency of luciferase, which catalyses the oxidation of d-luciferin to oxyluciferin [12]. The resulting light intensity (bioluminescence) is directly proportional to intracellular ATP concentration, which signals the metabolically active cells.

As mentioned, viability tests are well-established in cell lines as well as in in vitro models such as hepatic and intestinal slices [13]; however, investigations aimed at studying viability in the whole organism in the field of parasitology are currently scarce. In helminths, the ATP assay has been used in schistosomes [14], and the free-living nematode *Caenorhabditis elegans* [15], although to our knowledge it has not been performed in any gastrointestinal nematodes.

Therefore, in view of the need to obtain alternatives for the viability testing of *H. contortus*, we provide an assay that can assess not only infectious third-stage larvae (L3s), but also the adult stage, which causes haemonchosis. Toward this end, our efforts have been put forth within the optimization of the ATP bioluminescence assay on exsheathed third-stage larvae (xL3s) and ex vivo adult females and males of *H. contortus*.

## Materials and methods

### Parasite material

Third stage larvae (L3) of the susceptible MHco3 strain (Inbred Susceptible Edinburgh, ISE) [16] and multi-resistant MHco4 strain (White River, WR) [17] of *H. contortus* were obtained from the Institute of Parasitology, Slovak Academy of Science, Košice, Slovakia. Six-month-old sheep were orally infected with 8000 infective ISE L3s. Four weeks post-infection (pi), faecal samples were collected each day for one week. L3s were produced from eggs by incubating faeces in a plastic box covered with foil at 27 °C for 7 days [18], then the faeces were rinsed twice in tap water poured into conical measuring cups in which the larvae sank to the bottom. To remove dirt or dead individuals, the larvae were filtered through a 20 µm sieve (27 °C). Clean L3s at a concentration of ~2000–4000 L3 per mL were stored in culture flasks in water at 10 °C for several months. The L3s were exsheathed by incubation in water with 0.15% (v/v) sodium hypochlorite (NaClO) at 37 °C for 20 min. Then, the exsheathed L3s (xL3s) were washed five times in water by centrifugation at 1700 rpm for 3 min.

ISE adults of *H. contortus* were harvested from sheep abomasum 6 weeks pi using an agar method [19]. Using soft entomology forceps, males and females were separated based on morphological differences. All experimental procedures were examined and approved by the Ethics Committee of the Ministry of Education, Youth and Sports (Protocol MSMT-25908/2014-9).

### Optimization of the ATP assay

To prevent ATP degradation, all the following procedures except for the bioluminescence measurement itself were conducted on ice. An ATP bioluminescence assay was performed according to the manufacturer’s protocol (ATP Bioluminescence Assay Kit CLS II, Roche, Mannheim, Germany) with the following adjustments.

Homogenization was performed using a mix of zircon beads (sizes of 1.0 mm: 1.4 mm: 2.0 mm in 1:1:0.5 ratio, ~0.75 g) with the FastPrep-24 5G Instrument (MP Biomedicals, Santa Ana CA, USA). To remove cell debris, the homogenized samples were centrifuged at 4 °C using the Heraeus Biofuge Stratos (#3332 rotor, Thermo Fisher Scientific, Germany). For the ATP assay, 100 mM Tris(hydroxymethyl)aminomethane hydrochloride (Tris–HCl) with 2 mM EDTA, pH 7.8 denoted as “Tris/EDTA buffer”, was used.

The optimization steps were executed as follows: (i) the ATP standard curve was optimized for the lowest amount of biological material and in concordance to the working range of the kit, which is between 10^−6^ and 10^−11^ M ATP. In the xL3, we tested 50–500 larvae, while in the adults we tested 1–5 females or 2–10 males (males are approx. twice as small as females); (ii) the homogenizing steps (20 s, 8.0 m/s speed) were optimized for the number of cycles (1–5 cycles), (iii) the centrifugation was optimized for time (5 min, 10 min, 20 min) and speed (13 200 rpm, 16 000 rpm). The optimization steps (ii) and (iii) were performed using one female and two males in biological triplicates; (iv) the difference was measured between the incubation of the xL3s in different ratios of water and Tris/EDTA buffer.

ATP content was measured in a flat bottom black 96-well plate (Corning Costar, CLS9102, Sigma-Aldrich). The calibration curve was prepared by titration of the ATP standard from 0 to 1650 nM ATP. 50 µL for each calibration point in duplicates were pipetted into the wells. For the samples, 5 µL of supernatant and 45 µL of Tris/EDTA buffer were pipetted in duplicates. 5 µL of sample blank was prepared by mixing 50 µL EtOH/EDTA and 450 µL Tris/EDTA buffer, after which 50 µL of luciferase was added using an automatic dispensing pipette, with the luminescence measured immediately.

The bioluminescence readings of the ATP standard calibration curve and samples were performed with the plate reader Tecan Spark (Tecan Group Ltd., Switzerland) in room temperature in a kinetic method with 1 min cycles for 6 cycles in total. The stabilized values of relative luminescence units (RLU) from the final cycle were used for the quantification of ATP concentration.

### Optimization of protein assay

To eliminate variability in worm size in the adults, the ATP concentration was related to µg/mL of total proteins in a sample pellet. The protein was measured in technical duplicates per sample using bicinchoninic acid based on the manufacturer’s protocol (Pierce™ BCA Protein Assay Kit, ThermoScientific) with the following adjustments. To validate that the protein concentration positively correlated with the weight of worms, one female worm (*n* = 106) after incubation was washed in cooled phosphate-buffered saline (PBS), dried over a cellulose swab and its mass was measured on analytical balances (Sartorius CP225D).

After the ATP assay, the samples were evaporated using a centrifugal vacuum concentrator (Eppendorf Concentrator Plus) for 9 h, 45 °C. In addition to the samples, two extra tubes with 50 µL EtOH/EDTA + 450 µL Tris/EDTA were evaporated and served for the standard curve titration.

After evaporation, the sample pellets were dissolved in 100 µL of 5 M NaOH for 1 h, 800 rpm at 37 °C in ThermoMixer Comfort (Eppendorf). After the base hydrolysis, the samples were diluted with distilled water to 1 M NaOH. The same procedure of base hydrolysis was used with the final concentration of 0.5 M NaOH in the same total volume.

A series of dilutions of a common protein of the bovine serum albumin (BSA) were prepared by titration of 0.1% BSA in 1 M NaOH using solutions from two extra tubes. The pellet from the first extra tube was dissolved in 250 µL 2 M NaOH with 250 µL of a stock 0.2% BSA, and the pellet from the second extra tube was dissolved in 500 µL 1 M NaOH. This step ensured that the samples and calibration curve had a similar content of remaining salt from Tris/EDTA buffer. The absorbance of the blank background was then subtracted from the samples’ absorbances during the data evaluation.

### Quantification of ATP level in the mix of living and dead xL3s

To establish a relation between the ATP concentration and number of living larvae, we prepared samples of mixed viable and dead xL3s in different proportions so that the total number of larvae was 400 xL3s in 200 µL of water. The assay (using the already optimized conditions) was repeated three times.

### Viability of *H. contortus* xL3 and adults in time

For this experiment, we measured ATP concentration in the xL3s and adults for 7 days, and 5 days, respectively, using the already optimized conditions for ATP assay. The worms were incubated with 0.1% DMSO.

### Incubation of ISE and WR xL3 with anthelmintics

400 xL3s of susceptible ISE or the multi-resistant WR strain per sample were incubated in screw-top 2 mL micro tubes in 200 µL water with levamisole (LEV) in concentrations of 0.1 µM, 1 µM, 5 µM, 10 µM using 0.1% DMSO as negative control. Four replicates were prepared. After incubation for 24 h, 48 h, 72 h and 96 h, the samples were snap frozen using dry ice and stored immediately in −80 °C until further analyses. The mix of zircon beads was added to the frozen samples, which were homogenized for 20 s, 9.0 m/s speed and measured for ATP content.

### Incubation of ex vivo adults with anthelmintics

The adult worms were incubated ex vivo in the sterile “Hco medium” prepared by supplementation of RPMI 1640 medium (Sigma-Aldrich) so that it contained 0.8% glucose, 0.25 µg/mL amphotericin B, 10 U/mL penicillin, 10 g µg/mL streptomycin and 10 mM *N*-[2-hydroxyethyl]piperazine-*N*′-[4-butanesulfonic acid] (HEPES) buffer at pH 6.8 [20].

In one well, four females or eight males per sample were incubated with 900 µL of supplemented Hco medium in a 24-well plate (flat bottom TPP, Sigma-Aldrich) for 48 h, 37 °C, 5% CO_2_ with LEV or MOP in concentrations of 0.1 µM, 1 µM, 5 µM, 10 µM using 0.1% DMSO as the negative control. After the incubation time, to prevent the Hco medium from interfering with the BCA assay, the worms were rinsed in cooled sterile PBS (pH 7.4) on Petri dishes and separated into a screw-top 2 mL micro tube with 50 µL of cooled EtOH/EDTA by 1 female and/or 2 males, hence preparing four biological replicates. The samples were snap frozen using dry ice and stored immediately in −80 °C until further analyses. The frozen samples were then processed by adding 450 µL of cooled Tris/EDTA buffer and a mix of zircon beads, with the samples homogenized for 20 s, 8.0 m/s speed and measured for the ATP content.

### Calculations and statistics

All experiments were repeated three times except for the adult experiments, which were not repeated. For the standard curve data, the raw RLU (blank subtracted) and ATP standard concentration were log transformed. The concentration of ATP was calculated from the linear regression equation. Similarly, after blank subtraction, concentration of protein was calculated from the 0.1% BSA in a 1 M NaOH calibration curve. The linear regression equation and R-squared value were calculated with Microsoft Excel (version 2007). The results of ATP concentration (related to the amount of protein in adults) were normalized to control (0.1% DMSO), which represented 100% viability. The statistical significance was calculated in GraphPad Prism 9 using an unpaired Student’s *t*-test or multiple comparisons one-way ANOVA with Dunnett multiple comparisons test.

## Results

### Optimization of ATP assay

**Figure 1:**
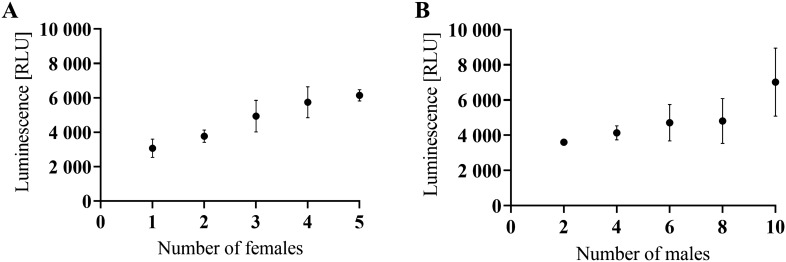
**Assessing of minimum number of adult worms.** The luminescence signal (RLU = relative luminescence unit) in relation to the growing number of (**A**) females or (**B**) males. Data represent the mean ± SD of three replicates for each point.

The results from the optimization steps are as follows:

i. The minimum optimal number of larvae/adults for the ATP assay and subsequent BCA assay was assessed. In the xL3 experiments, the luminescence signal was detectable even for 50 xL3s; however, the following experiments resulted in the optimal use of 400 xL3 in 200 µL. In the adults, the luminescence signal was detectable and optimal even at one female or two males per sample (Figure 1). The working range during our experiments was from 1.65 nM to 1650 nM ATP. The generated luminescent signal increased as the number of adults increased, however, it was not in direct proportion to the number of females or males. Surprisingly, the luminescence signal in the sample with five females did not grow at a similar rate as samples with fewer females. Further experiments showed that the addition of more than 5 µL of homogenate into the reaction did not increase the luminescent signal. Based on the comparison of estimated (calculated) values and real values, we assume the presence of quenchers of luminescence signal in the homogenate. We calculated the lower and upper confidence intervals on a 95% level of significance for each sample observing that the real values were not within the estimated range, but always lower (Additional file 1).
ii. One homogenization cycle was sufficient to release the ATP from the cells. Further homogenization cycles did not yield a higher concentration of ATP (Additional file 2A).
iii. The centrifugation speed did not make any significant difference between 13 200 and 16 000 rpm. In further experiments, the homogenized samples were centrifuged at 13 200 rpm for 10 min (Additional files 2B, C).
iv. The luminescence signal was the highest for the xL3s in water only (Additional file 2D).

**Figure 2:**
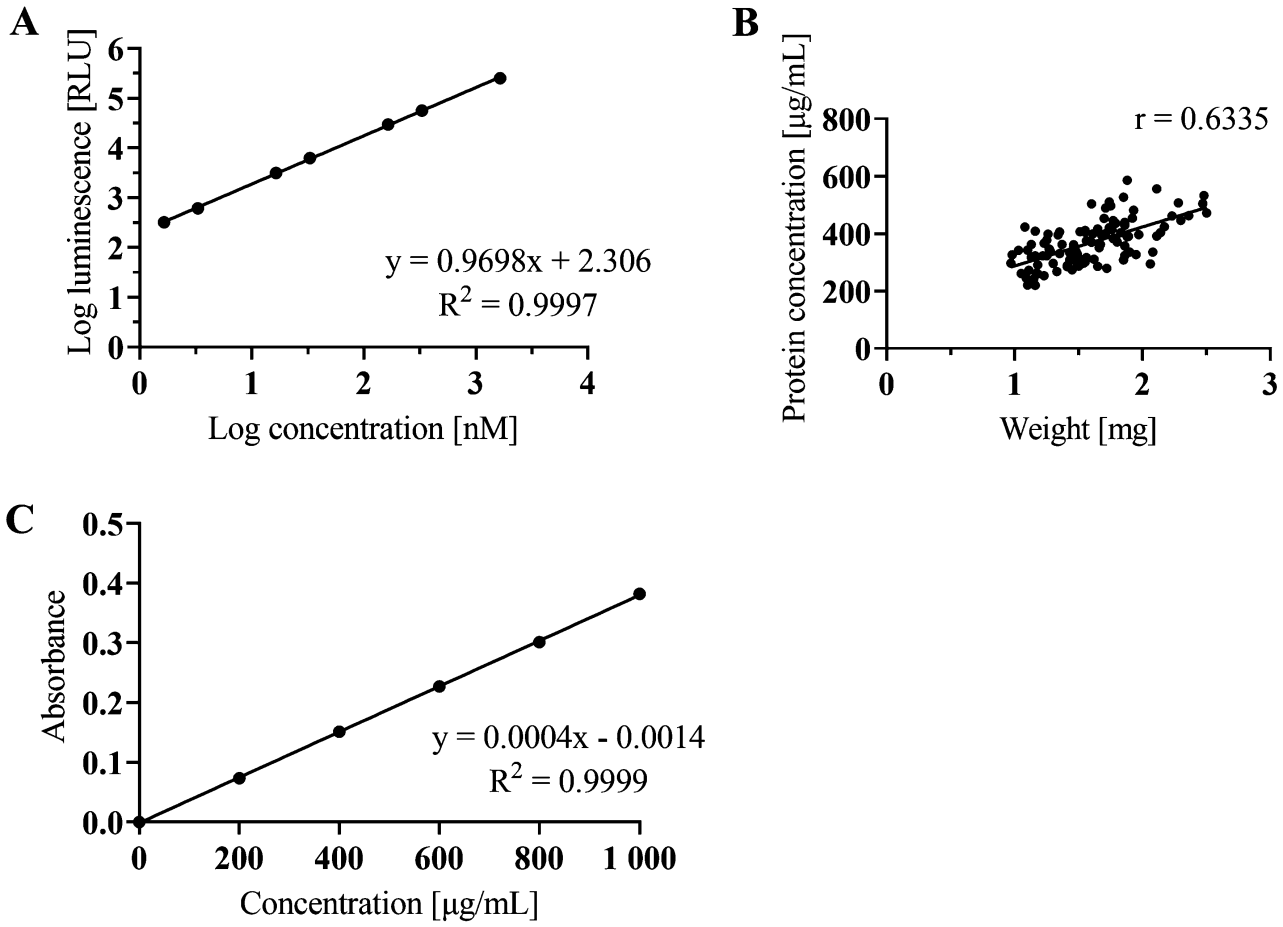
**Optimization of the ATP and BCA assay.A** Standard curve for ATP assay, each point is the average of technical duplicates. **B** Positive correlation between protein concentration and weight of one female with Pearson correlation coefficient r. **C** Standard curve for BCA assay, each point is the average of technical tetraplicates.

The calibration curve for ATP standard is shown in Figure 2 (Figure 2A). The luminescence of the chemical blank (50 µL EtOH/EDTA + 450 µL Tris/EDTA) did not vary from the luminescence of the blank from the calibration curve (Tris/EDTA), therefore it was not pipetted on the plate in the other experiments.

### Optimization of the protein assay using bicinchoninic acid

Due to the various sizes of the females, we measured protein concentration to derive the correct normalization of the luminescence signal, with the protein concentration positively correlating to the mass of the worm (r = 0.6335, R^2^ = 0.4013, *P* (two-tailed) < 0.0001)) (Figure 2B). The standard deviation of protein concentration and mass of worm in the biological replicates were also similar (20.9% and 22.6% from the mean, respectively). For the workflow, we used correction to protein for further experiments in adults.

For protein hydrolysis we used a strong alkaline 5 M NaOH solution. To bring about compatibility with the BCA protein assay, we diluted the samples to 1 M NaOH. A measurement of 1 M NaOH yielded a higher protein concentration than did 0.5 M NaOH with statistical significance *P* < 0.05 for all samples except sample F3. The calibration curves for both 0.5 M and 1 M NaOH are also presented (Additional file 3). The example of the calibration curve of 0.1% BSA is shown in Figure 2C.

### Quantification of ATP level in the mix of living and dead xL3s

**Figure 3:**
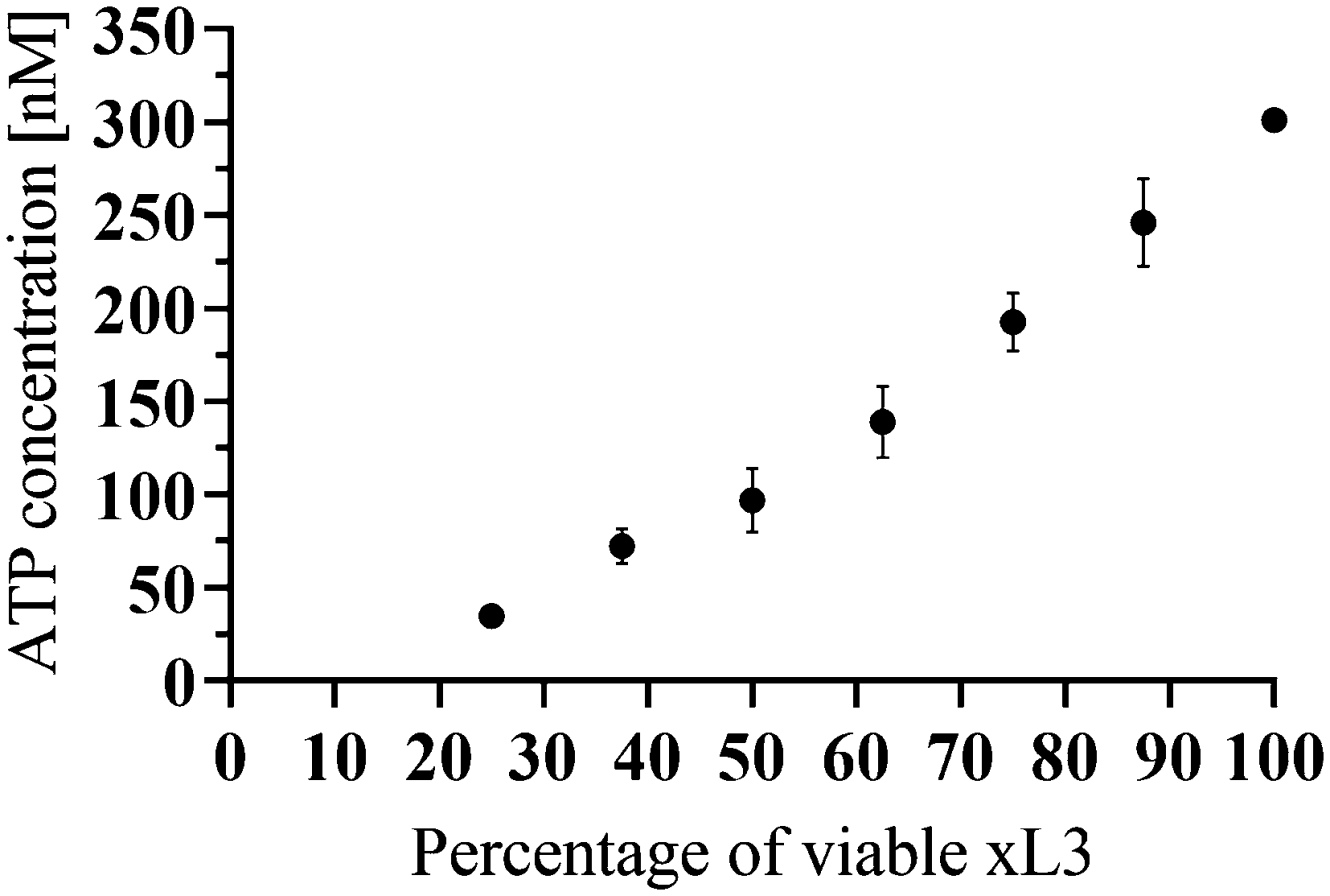
**Viable and dead xL3s.** Relation of ATP concentration to number of viable xL3s from a total mixture sample of dead and viable xL3s of 400 xL3 in 200 µL. The data represent mean ± SD.

The concentration of ATP was also influenced by the proportion of dead larvae in the sample. However, with an increasing proportion of dead to living larvae, the ATP signal became lower. The decline was so rapid that almost no ATP was detected when 25% of larvae were still alive (Figure 3).

### Viability of *H. contortus* xL3 and adults in time

**Figure 4:**
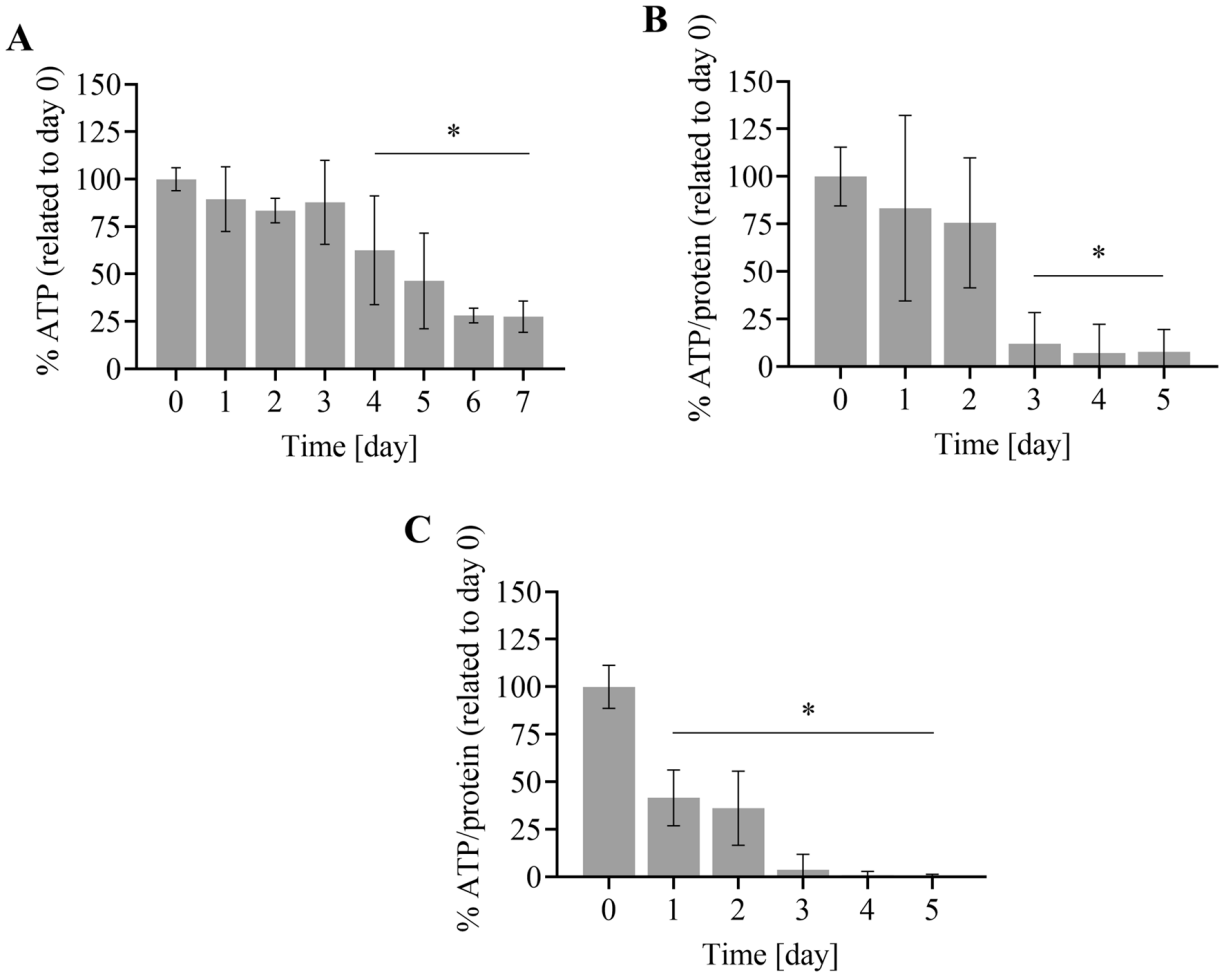
**The ATP content in time.** The ATP concentration was measured in consequent days in (**A**) xL3s, and in ex vivo (**B**) females (**C**) and males. The asterisk denotes statistical significance at *P* < 0.05 in comparison to day 0.

The xL3 viability was measured daily for 7 days, with the content of ATP decreasing at day 4, 5, 6 and 7 compared with day 0 (*P* < 0.05, one-way ANOVA multiple comparisons) (Figure 4A). Based on these results, we incubated the xL3s with the anthelmintics up to 96 h.

The viability of ex vivo adults was measured over five consecutive days. In the females, the ATP content at day 1 and day 2 remained high, and not significantly different from day 0. A significant decrease in ATP content in the females was observed at day 3 (Figure 4B). In the males, a significant ATP decrease was found at day 1 compared to day 0, but the viability remained the same at day 2, then decreased substantially at day 3 (Figure 4C). Based on these results, we incubated the adults with anthelmintics for 48 h.

### The use of the ATP assay for the assessment of the effect of levamisole on ISE and WR xL3 viability

**Figure 5:**
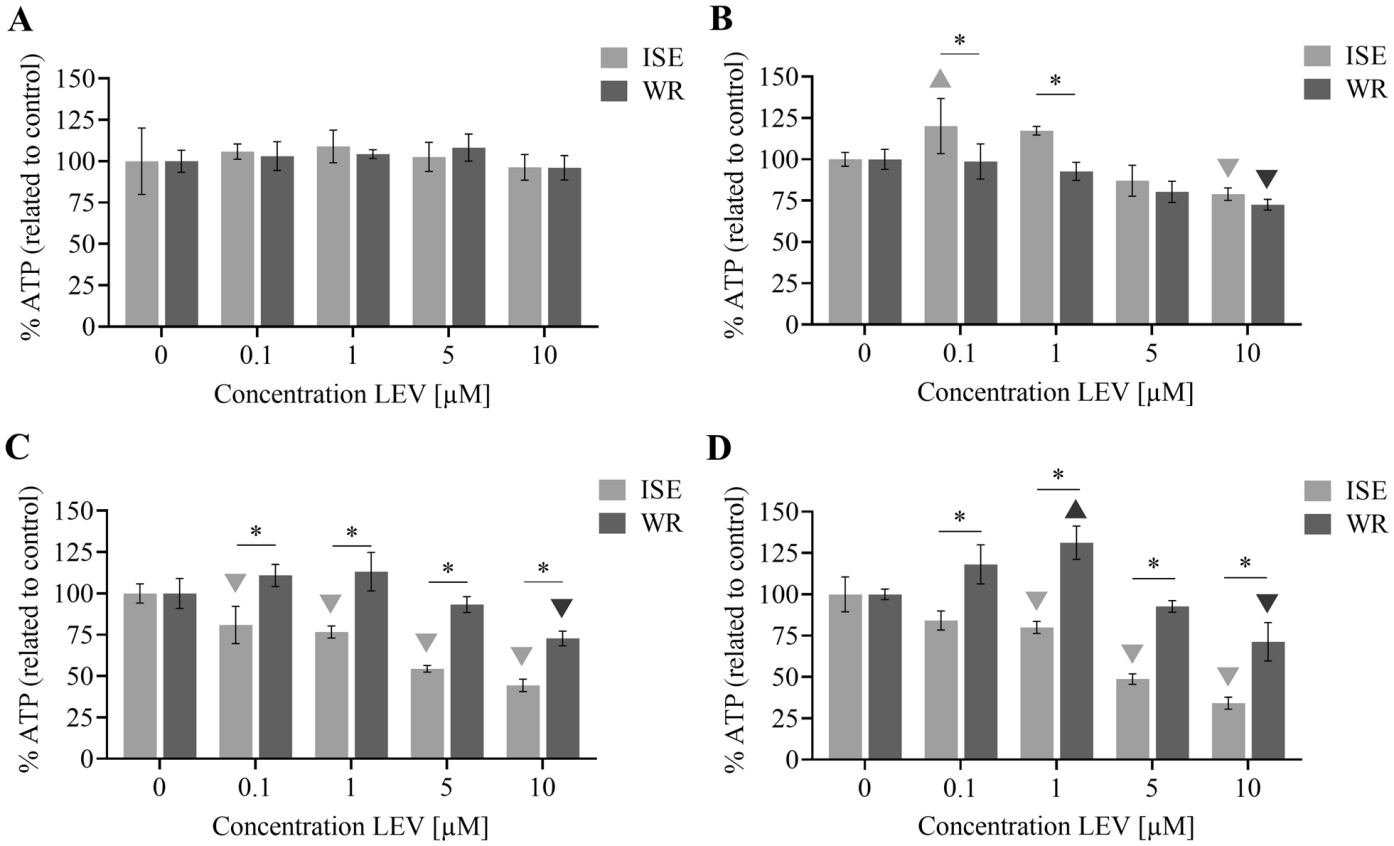
**The effect of levamisole (LEV) on viability of xL3 in susceptible ISE and resistant WR strain.** xL3s were incubated with different concentrations of LEV for (**A**) 24 h, (**B**) 48 h, (**C**) 72 h, (**D**) 96 h. The ATP level from each day was compared to the 0.1% DMSO. The data represent mean ± SD, downward filled triangle. Filled triangle denote statistically significant decrease or increase, respectively, within the ISE or WR strain, whereas asterisk denotes statistical difference between ISE and WR strain (*P* < 0.05).

When we incubated the ISE and WR xL3 with levamisole (LEV) at 0.1–10 µM concentration (pre-dissolved in DMSO) up to 96 h (Figure 5), the level of ATP was not different from the control (with DMSO only) after 24 h, neither in the ISE nor in the WR strain. After 48 h, the ATP concentration increased in the ISE xL3 incubated with 0.1 µM LEV, whereas it significantly decreased at 10 µM LEV. In the ISE xL3, we observed a significant concentration-dependent decrease in ATP level at 72 h and 96 h. On the contrary, in the WR xL3, the level of ATP significantly decreased only at the highest concentration of 10 µM. Also, in the WR xL3, the ATP concentration increased in the xL3 incubated with 1 µM LEV after 96 h. Overall, a significant difference between the ISE and WR strain was observed at 72 and 96 h treatments, with the ISE exhibiting reduced viability in all concentrations.

### The use of the ATP assay for the assessment of the effect of levamisole and monepantel on adult viability

**Figure 6:**
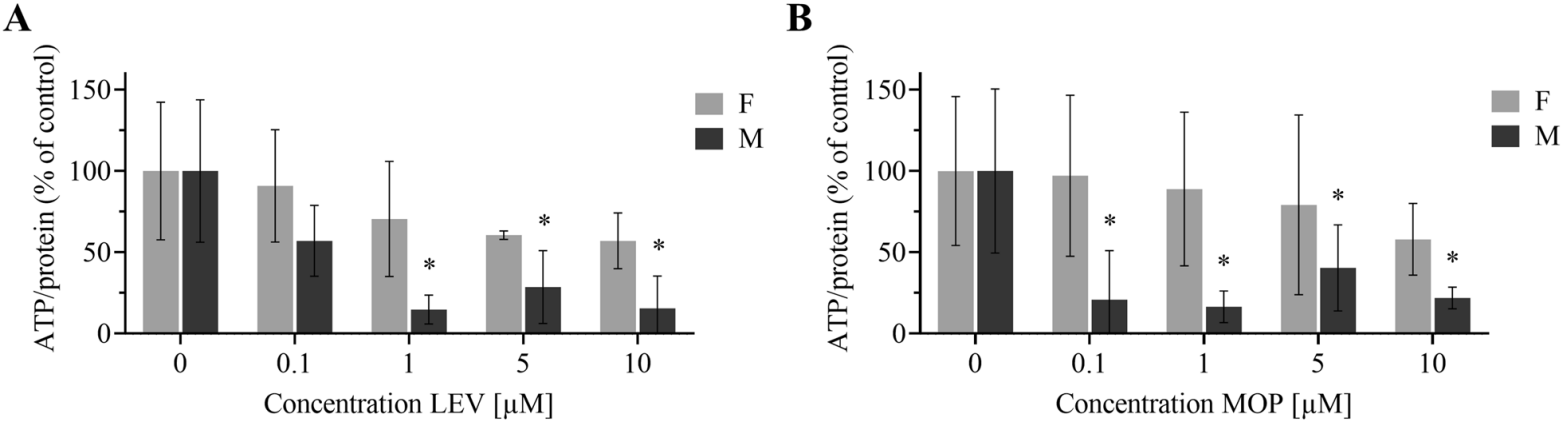
**ATP in adult females and males treated with levamisole and monepantel.** Levels of ATP/protein relative to control (0.1% DMSO) in females (F) and males (M) after 48 h incubation with (**A**) levamisole (**B**) monepantel. The data represent mean ± SD; asterisk denotes statistical significance at *P* < 0.05.

We incubated males and females of the ISE strain with LEV or MOP at concentration 0.1–10 µM for 48 h (Figure 6), following which the ATP content was assayed using the optimized method. Although huge interindividual differences in ATP/protein in the biological replicates were detected, the content of ATP/protein decreased significantly in the males incubated with 1 µM, 5 µM and 10 µM LEV. A significant decrease was also observed in the males incubated with all concentrations of MOP. However, only an insignificant drug-mediated decrease in ATP level was found in the females.

## Discussion

The gastrointestinal nematode *H. contortus* is often used for the screening of potential drug toxicity and drug resistance [2], although classical developmental methods are not optimal in terms of the laborious evaluation needed as well as the use being relegated to the early developmental stages only. Our objective was to establish a method that does not require microscopical manual counting and, mainly, that will be applicable to later developmental stages, i.e. xL3 and adults. We searched for a marker that would distinguish between dead and alive individuals as well as which could be quantifiable. We focused on adenosine triphosphate (ATP), as the maintenance of an appropriate ATP level is crucial for all cells and cellular organisms. Moreover, the ATP bioluminescent assay is a widely used method for measuring the viability and/or energy metabolism in various cell lines and tissue slices. In *H. contortus,* the ATP metabolism and differences between the larval and adult stages has been described [21, 22], but ATP quantification as a viability marker has not been tested yet.

Keeping all this in mind, we wanted to determine if the ATP bioluminescent assay is a suitable tool to assess the viability of the parasitic nematode *H. contortus.* If so, we would seek to optimize the entire procedure. The initial ATP protocol was taken from the study of precision-cut liver and intestinal slices [13], since ex vivo explants of tissues are close to the size and complexity of our parasite, in contrast to cell lines.

For experiments with the xL3s, we opted for measuring 400 xL3s in water instead of the common Luria Bertani medium or Tris/EDTA buffer. Comparisons of the data related to mg of protein along with data related only to number of larvae show no significant differences (data not shown). Based on these findings, it was not necessary to measure the protein concentration in the xL3, although a higher number of replicates (6–8) for each sample is strongly recommended.

Concerning the adults, a sufficient ATP signal was produced using only one female or two males. On the contrary, a relatively high number of replicates is essential due to the huge interindividual differences in ATP level in *H. contortus* adults. It is well described that various cell types have different amounts of ATP, and the values reported for ATP level in cells vary considerably [23]. As ATP level strongly depends on the size of organism, the normalization of the raw data obtained in adults to total protein concentration is necessary. Despite this, the interindividual variability in ATP level in the adults remained considerable. A complex of biochemical compositions, such as salts, organic small molecules, proteins (lysing enzymes) might cause a series of issues, including the enzymatic degradation of ATP as well as signal interference. This disadvantage can be partially overcome by using a high number of replicates.

The optimized protocol for xL3 of *H. contortus* was applied to test the LEV effect on larvae from the ISE strain (drug susceptible) and the WR strain (drug resistant). A significant difference in the viability of the ISE and WR strain was observed, indicating that the ATP assay might serve well for the detection of drug-resistant isolates. As incubation with LEV for 72 h and longer decreased the level of ATP in a concentration-dependent manner in ISE xL3; this assay can be used to test potential anthelmintically active compounds. However, the non-proportional decrease and big interindividual differences make the accurate determination of half inhibitory concentration values (IC_50_) impossible. The ATP level is modulated by many factors, and an elevation in ATP can also reflect a stress response [24]. We therefore hypothesize that 0.1 µM LEV did not kill the ISE larvae in 48 h, but did evoke certain stress defence responses. A similarly increased ATP level was observed in 1 µM LEV in WR xL3 in 96 h, indicating the propensity of the resistant strain to survive with higher concentrations of drug. Simultaneously to the ATP assay, the phenotype of xL3s was examined. We observed an overt inhibition of motility (coiled phenotype) in ISE xL3 even after 24 h 5 µM LEV, although ATP level did not significantly decrease. We propose that worms react to the compound by accelerating their metabolism, and it takes time for ATP to stabilise or eventually decrease. In one study of spermatozoa motility and viability, the viability (live, dying, dead spermatozoa) was not the main factor controlling their movement, and ATP content was not involved in controlling the end of spermatozoa motility [25]; these findings were also observed in our experiments. Although there have been many reports of drugs reducing the motility of different larval stages (xL3s or L4) of *H. contortus* [26–28], causality in terms of the immobile phenotype in viable larvae has not been documented.

The optimized protocol for *H. contortus* adults was used to test the effect of LEV and MOP on females and males. Two anthelmintics, each with different modes of action, were chosen: LEV works on the parasite nervous system as a nicotinic agonist and causes paralysis in nematodes, whereas the MOP mechanism is connected with acetylcholine receptor subunits [29]. In incubating the males with both LEV and MOP, we observed a significant dose-dependent reduction in ATP level. In the females, the anthelmintics-mediated decrease of ATP level was milder and insignificant due to huge inter-individual variation. Although it is known that females metabolize (deactivate) drugs more extensively than males [30, 31], the low sensitivity of the adult females to LEV and MOP was surprising, and the reason for this remains unclear. The bioluminescent ATP assay performed in males, however, may be a suitable method for testing potential drug effects in *H. contortus* adults.

Nevertheless, although in terms of high-throughput methods, the ATP assay is not ideal for the primary screening of large libraries of compounds, it can be useful for the secondary screening of hit compounds or for the drug repurposing of compounds that target the electron transport chain and phosphorylation reaction, thus reducing ATP synthesis. Because of the complexity of an organism-level system as well as interindividual variability, the ATP assay can be challenging, and proper sample management and optimization procedures will inevitably cause minor or major difficulties as the assay is applied in different studies. Moreover, ex vivo studies on these worm stages are limited by the inefficient capability of maintaining hematophagous worms in an artificial culture media for a long time. It is also important to have proper controls to relate and compare the results to.

Finally, it is clear that the assessment of viability via the measurement of ATP concentration is quantitative and it does not require microscopy. Furthermore, to our knowledge this is the only available biochemical method targeting the parasitic stage of the worm which causes haemonchosis, i.e. adult worms. The advantage of this method is its sensitivity, hence the low amount of biological material, although 6–8 biological replicates are necessary. This method can be used as a complementary assay for the phenotypic screening of new potential anthelmintics in exsheathed third-stage larvae and in adult males. Additionally, ATP assay might serve for the detection of drug-resistant isolates. Nevertheless, it is also important to take into account the fact that resistant strains contain a mixture of susceptible and resistant isolates hence the ATP values in individual adults can also vary considerably.

## Supplementary Information

**Additional file 1:Table of measured and theoretical ATP concentrations.** The table of ATP concentration in relation to the increasing volume of biological homogenate. The measured ATP concentration was lower than the theoretical values.

**Additional file 2: Optimization of ATP assay.** Optimization of (A) number of homogenization cycles, (B) time and (C) speed of centrifugation in female and male adults. (D) The ATP signal (RLU = relative luminescence unit) in xL3 samples (*n* = 3) incubated at different ratios of water and Tris/EDTA buffer. The luminescence signal of all mixtures of water with buffer was related to the luminescence signal of water only, * *P* < 0.05.

**Additional file 3**: **Comparison of 0.5 M NaOH and 1 M NaOH.** (A) Standard curves for 0.1% BSA in 0.5 M NaOH and 1 M NaOH, where each point represents mean of technical tetraplicates ± SD. The equation for 0.5 M NaOH was y = 0.0004x + 0.0086 (R^2^ = 0.9979), for 1 M NaOH was y = 0.0007x + 0.019 (R^2^ = 0.9948). (B) Comparison of protein concentration in female (F) and male (M) in 1 M or 0.5 M NaOH, the number denotes number of worms (e.g. F3 means three female worms). The data represent mean of three biological replicates ± SD. * denotes statistical significance at *P* < 0.05.

## Data Availability

The datasets supporting the conclusions of this article are included within the article text and additional files.

## Abbreviations

ATP: Adenosine triphosphate
BCA: Bicinchoninic acid
BSA: Bovine serum albumin
DMSO: Dimethyl sulfoxide
EDTA: Ethylenediaminetetraacetic acid
ISE: Inbred susceptible-Edinburgh strain (MHco3)
LEV: Levamisole
MOP: Monepantel
PBS: Phosphate-buffered saline
RLU: Relative luminescence unit
Tris–HCl: Tris(hydroxymethyl)aminomethane hydrochloride
WR: White River strain (MHco4)
