# Supplementary material for: The ATP bioluminescence assay: a new application and optimization for viability testing in the parasitic nematode Haemonchus contortus
Source: Vet Res. 2021 Sep 30;52:124. doi: 10.1186/s13567-021-00980-4 (PMC8482649; doi:10.1186/s13567-021-00980-4)
Supplement: Supplementary file 1 — Additional file 1:Table of measured and theoretical ATP concentrations. The table of ATP concentration in relation to the increasing volume of biological homogenate. The measured ATP concentration was lower than the theoretical values. [file 13567_2021_980_MOESM1_ESM.docx]

| **Volume**  **of homogenate [µL]** | **Volume**  **of Tris/EDTA [µL]** | **ATP concentration [nM]** | | |
| --- | --- | --- | --- | --- |
|  |  | Measured | Theoretical | Per 1 µL of homogenate |
| 5 | 45 | 820.84 | 820.84 | 164.17 |
| 10 | 40 | 512.68 | 1641.67 | 51.27 |
| 20 | 30 | 349.34 | 3283.34 | 17.47 |
| 50 | 0 | 301.38 | 8208.36 | 6.03 |
