# Supplementary figures and images for: The ATP bioluminescence assay: a new application and optimization for viability testing in the parasitic nematode Haemonchus contortus
Source: Vet Res. 2021 Sep 30;52:124. doi: 10.1186/s13567-021-00980-4 (PMC8482649; doi:10.1186/s13567-021-00980-4)

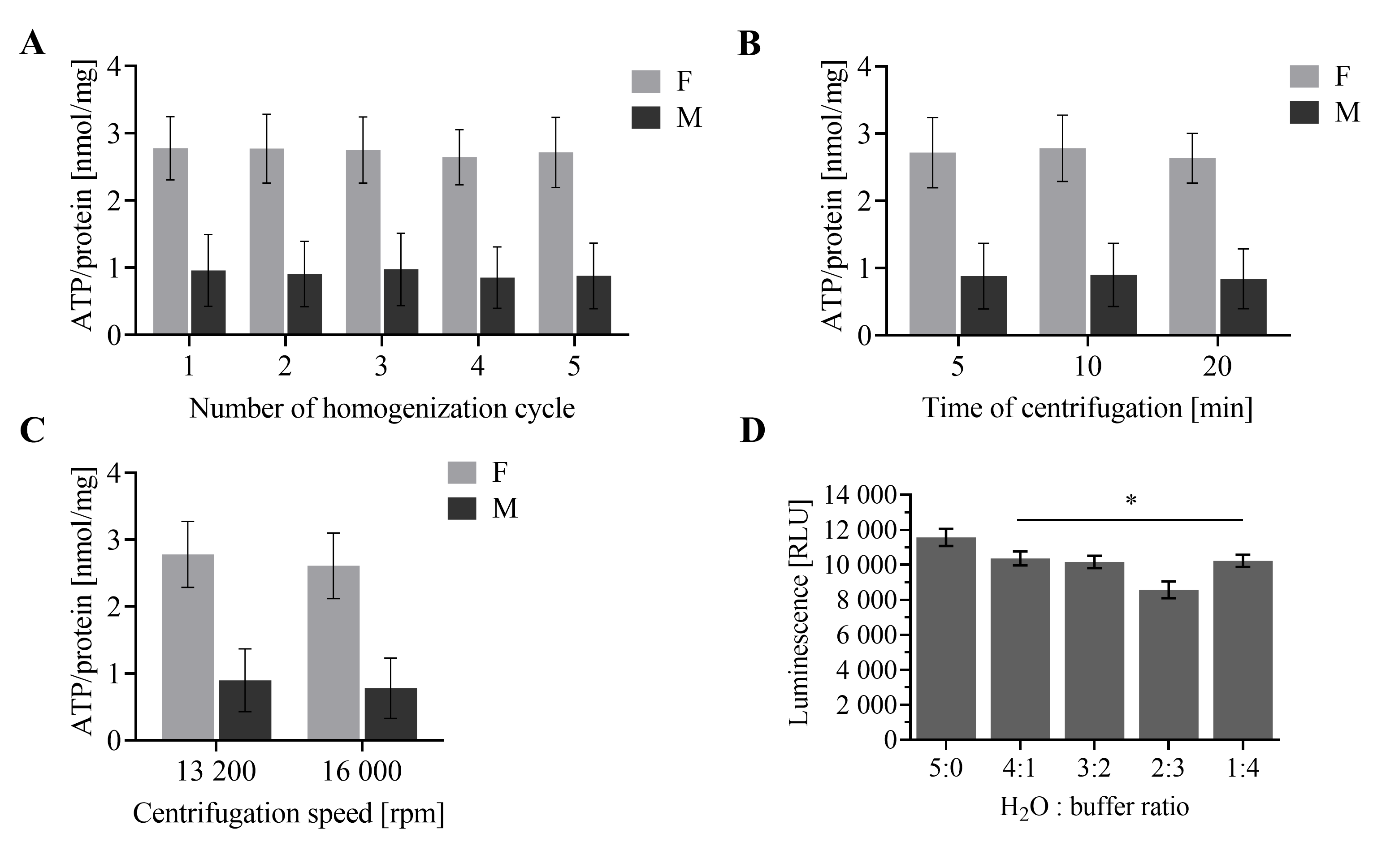

Supplement: Supplementary file 2 — Additional file 2: Optimization of ATP assay. Optimization of (A) number of homogenization cycles, (B) time and (C) speed of centrifugation in female and male adults. (D) The ATP signal (RLU = relative luminescence unit) in xL3 samples (n = 3) incubated at different ratios of water and Tris/EDTA buffer. The luminescence signal of all mixtures of water with buffer was related to the luminescence signal of water only, * P < 0.05. [file 13567_2021_980_MOESM2_ESM.tif]

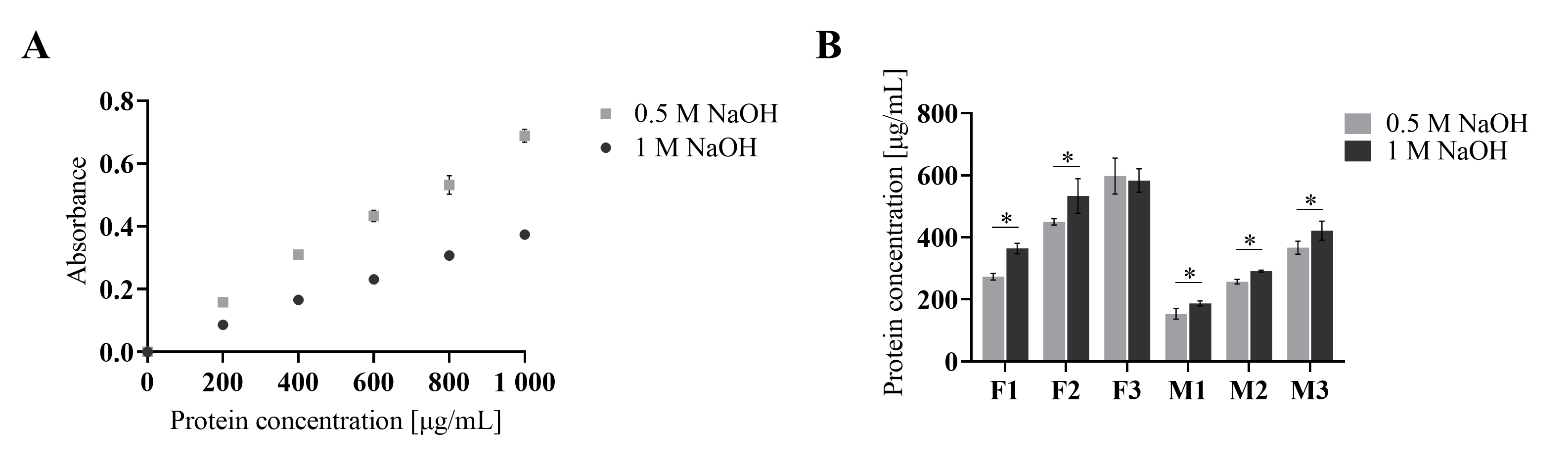

Supplement: Supplementary file 3 — Additional file 3: Comparison of 0.5 M NaOH and 1 M NaOH. (A) Standard curves for 0.1% BSA in 0.5 M NaOH and 1 M NaOH, where each point represents mean of technical tetraplicates ± SD. The equation for 0.5 M NaOH was y = 0.0004x + 0.0086 (R2 = 0.9979), for 1 M NaOH was y = 0.0007x + 0.019 (R2 = 0.9948). (B) Comparison of protein concentration in female (F) and male (M) in 1 M or 0.5 M NaOH, the number denotes number of worms (e.g. F3 means three female worms). The data represent mean of three biological replicates ± SD. * denotes statistical significance at P < 0.05. [file 13567_2021_980_MOESM3_ESM.tif]
